# An Application of the Gaussian Plume Model to Localization of an Indoor Gas Source with a Mobile Robot

**DOI:** 10.3390/s18124375

**Published:** 2018-12-11

**Authors:** Jorge Edwin Sánchez-Sosa, Juan Castillo-Mixcóatl, Georgina Beltrán-Pérez, Severino Muñoz-Aguirre

**Affiliations:** Facultad de Ciencias Físico-Matemáticas, Benemérita Universidad Autónoma de Puebla, Av. San Claudio y 18 Sur, Col. San Manuel, C.U., C.P. 72570 Puebla, México; jorge_edwin.ss@hotmail.com (J.E.S.-S.); jcastill@fcfm.buap.mx (J.C.-M.); gbeltran@fcfm.buap.mx (G.B.-P.)

**Keywords:** Gaussian plume model, gas source localization, concentration distribution, wind velocity distribution, metal-oxide semiconductor sensor, robotic system

## Abstract

The source localization of gas leaks is important to avoid any potential danger to the surroundings or the probable waste of resources. Currently there are several localization methods using robotic systems that try to find the origin of a gas plume. Many of these methods require wind velocity information involving the use of commercial anemometric systems which are extremely expensive compared to metal oxide gas sensors. This article proposes the validation of the Gaussian plume model inside an empty room and its application to localize the source of a gas plume without employing anemometric sensors, exclusively using concentration data. The model was selected due to its simplicity and since it easily admits variants closer to reality, explaining the behavior of pollutants transported by the wind. An artificial gas source was generated by a conventional fan and liquid ethanol as contaminant. We found that the physical fan, far from making the model impossible to implement, enriched the information and added realism. The use of a robotic system capable of autonomously mapping the room concentration distribution is described. The results showed that the Gaussian plume model is applicable to localize our experimental gas source. An estimated position of the source with a deviation of 14 cm (6.1%) was obtained.

## 1. Introduction

Gas source localization can be very useful in solving problems such as localizing fuel leaks in harmful environments, contamination sources, victims in accidents or fires in their early stages, among others. The need for mobile robots arises when the gas source is composed of toxic or explosive gases, when the gas source occurs in an inaccessible location, or when a continuous verification of the environment is necessary, etc. In this kind of studies, robots tend to be autonomous since it is desirable to avoid the implications of a human operator such as training, remuneration, recesses and mistakes; although teleoperated systems have also been exhibited [1]. Several studies that have estimated the source of gas plumes using autonomous mobile robots have been reported. From these studies it is possible to distinguish two ways of proceeding according to the navigation of the robots: (a) localization by tracking that involves real-time data for constant trajectory correction, based on *chemotaxis* using concentration gradients [2], based on *anemotaxis* using airflow gradients [3], or in most of cases based on combinations of *taxes* [4,5,6]; and (b) localization by mapping on a fixed trajectory collecting information on gas distribution and air flow and then estimating the source of the plume using statistical algorithms [7,8,9], a method known as *infotaxis* [7]. It is necessary to mention that these *infotaxis* methods are designed to obtain the gas distribution map (highlighting the DM + V Kernel [9]) with enough information to estimate the source position. A detailed review on the different use of strategies to localize gas sources is presented by Lilienthal et al. [10] and Monroy et al. [11]. Either by tracking or mapping, in most cases, information about wind direction and velocity is used. Both quantities are commonly measured using expensive anemometer-vane systems [4,5,6,7]. The localization problem is so complicated that even algorithms based on *chemotaxis* make use of wind direction as was shown by Russell et al. [2]. Osório et al. [12] reported an algorithm using only concentration data for the navigation of their robot (tracking), however their results were not satisfactory.

The use of anemometric systems may be adequate in the field of research since, due to the plumes complexity, it is important to collect as much information as possible. However, in various applications the current cost of a commercial anemometer could be a serious inconvenient, especially if mass production is considered. For instance, an anemometer used in this kind of work are the Young 81000 ultrasonic [7,9,13] ($2806 USD [14]), or the Gill Windsonic [12,15,16] ($1265 USD [17]) devices; contrasting with commercial metal-oxide sensors, such as TGS2620 ($10 USD) or MQ3 ($2 USD). In this paper we describe the experiences that led us to propose a procedure to localize a gas source without using wind information, employing only concentration data obtained from metal-oxide gas sensors using the Gaussian plume model making the corresponding assumptions about the environmental conditions to validate the model.

### 1.1. Gaussian Plume Indoor Application

Atmospheric dispersion models have been developed for several decades in order to understand the random and unpredictable plumes behavior of material thrown into the atmosphere. Due to its simplicity, the Gaussian plume model is frequently used; this kind of plume is generated by a point source with constant emission subjected to a unidirectional wind. These simplifications allow obtaining analytical solutions even introducing slight variations to the model to give it more realism for specific circumstances such as the vertical variation of velocity due to the atmospheric strata [18], the possible effects of absorption by the soil [19], or whether the plume occurs on a hot day or on a cold night [20].

At first sight, simplifications may seem as a limitation in real environments. Although the plume is instantaneously irregular and random, with a sufficiently long sampling the random behavior is exchanged for a uniform behavior around the average [19]. In this sense, Marjovi et al. [21] present the structure of a natural plume at different time scales evidencing different gas distribution maps in presence of the same phenomenon. In addition, although the wind in real environments cannot be considered laminar, in many cases under sufficient time it is possible to determine a preferred direction.

The Gaussian model is particularly effective in many situations where the main objective is to know the distance and concentration ranges of pollutants thrown to the atmosphere by chimneys [19,22]. Although it has also been applied in agriculture in the dissemination of pollen [23] and in entomology, trying to understand the behavior of insects in the presence of pheromones carried away by the wind [24]. A common aspect of these studies is the use of the theory in sources located in open areas where the plume can extend up to several kilometers [20,22] and the aforementioned simplifications are valid.

A different problem arises when the plume is confined inside an empty room, interacting in a short time with walls, soil and ceiling; also presenting strong effects of accumulation of the pollutant in the air. Even so, two papers related to the direct application of the Gaussian plume model indoors have been presented, one by Ishida et al. [5] using a nozzle to launch saturated ethanol vapor and another one by Marques et al. [6] using three fans in conjunction with liquid ethanol and methanol. However, it is not possible to appreciate the effectivity of the model itself owing to the following reasons:

- The model played a secondary role and the algorithms were enriched with wind velocity data.
- Their experiments were performed without paying attention to the validity of the model in these singular circumstances, specifically ignoring the distribution of velocities generated by their gas sources and the reduced dimensions of the rooms. For the case of Ishida et al., the concentration distribution in the immediate vicinity of their source is conical, approaching the turbulent jet theory [25] more than the Gaussian plume. For the case of Marques et al., they use two sources, which can provoke overlapping effects.
- Possible inaccuracies in the concentration when ignoring the effects of temperature and humidity on the gas sensors, in both cases metal-oxide sensors.

The model necessarily requires a non-zero wind velocity. In this research we use a gas source with an air flow larger than 0.1 m/s. The study of gas sources with natural wind (<0.1 m/s) requires a different methodology [9,13,26,27].

### 1.2. Objective

In this paper we want to show that a gas plume inside a room without obstacles under a prolonged sampling can be considered uniform and symmetrical similar to the Gaussian plume. Therefore, it will be possible to estimate the gas source position using such model.

We have proceeded with the following steps: (1) we modified the atmospheric dispersion model to fit the room circumstances; (2) based on the model, we obtained the mathematical expression that allows to estimate the source position; (3) experimentally, we obtained the wind velocity distribution generated by a commercial fan; (4) experimentally, knowing *a priori* the gas source position, we evaluated its concentration profile; (5) experimentally, we mapped the room using an autonomous robotic system without using information about the source location and without collecting information about the wind (direction or velocity), and we estimated the gas distribution in a plane parallel to the soil; (6) we discuss the results and present our conclusions.

## 2. Theoretical Gas Distribution

### 2.1. Gaussian Plume Model

The equation for a Gaussian plume, as a steady state solution of the advection-diffusion equation, is presented by several authors [6,19,20,24]:

(1) $$\zeta \left(x,y,z;h\right)=\frac{Q}{2\pi u\sigma_{y}\sigma_{z}}\exp\left(-\frac{y^{2}}{2\sigma_{y}^{2}}\right)\left\{\exp\left[-\frac{{\left(z-h\right)}^{2}}{2\sigma_{z}^{2}}\right]+\alpha \;\exp\left[-\frac{{\left(z+h\right)}^{2}}{2\sigma_{z}^{2}}\right]\right\}$$

where ζ is the pollutant concentration [kg/m^3^] at a point (x,y,z) [m], with the x-axis aligned to the wind direction, the y-axis aligned to cross-wind, and the z-axis aligned to the height above soil; Q is the gas emission ratio (constant) [kg/s] of the punctual source located in (0,0,h) with h the height above the soil [m], $\sigma_{y}\left(x\right)$ and $\sigma_{z}\left(x\right)$ are the coefficients of diffusion [m], u is the wind velocity (constant) [m/s], and α is the proportion of material reflected back into the plume when it reaches soil level [24] (for perfectly absorbing soil α=−1 [22] while for perfectly reflecting soil α=1 [19,22]). We consider the following particular characteristics based upon our experimental conditions:

(1) The contaminant is emitted from soil level, thus h=0;
(2) The concentration is measured at a constant height $z=z_{0}$;
(3) The environmental conditions are stable inside the room, therefore the diffusion is considered isotropic so that $\sigma_{y}=\sigma_{z}=\sigma$;
(4) The contaminant does not penetrate the soil, accordingly α=1;

then Equation (1) becomes:

(2) $$\zeta \left(x,y\right)=\frac{Q}{\pi u\sigma^{2}}\exp\left(-\frac{y^{2}+z_{0}{}^{2}}{2\sigma^{2}}\right).$$

In summary, Equation (2) considers that the emission ratio is constant, the wind velocity is constant and unidirectional, the environmental conditions are stable, the gas source is at ground level, the measurement height is constant and the soil is totally reflecting.

The diffusion coefficient σ is quite important in practical applications. Taylor [28] deduces the form of σ by distinguishing two dispersion behaviors that are dependent on the correlation between the initial and final state of movement of the particles that constitute the pollutant. When the correlation is high, the plume presents a conical behavior as generated by a nozzle [25] with σ proportional to time t. If the correlation is low, then the plume can approximate the Gaussian model with $\sigma \propto t^{1/2}$. The last correlation governs our source. Therefore, considering a constant wind velocity:

(3) $$\sigma^{2}=Kt=\frac{K}{u}x,$$

where K is a proportionality constant known as the turbulent diffusion coefficient (eddy diffusion coefficient) [5,19,22]. Marques et al. [6] have also applied the same form $\sigma =ax^{b}$ in their indoor/localization study. Substituting Equation (3) in Equation (2):

(4) $$\zeta \left(x,y\right)=\frac{Q}{\pi Kx}\exp\left[-\frac{u\left(y^{2}+z_{0}{}^{2}\right)}{2Kx}\right].$$

For convenience, we propose to write Equation (4) in terms of the maximum concentration $\zeta_{\max}=\frac{2Q}{\pi uez_{0}{}^{2}}$ located along the x-axis at distance $x_{\max}=\frac{uz_{0}{}^{2}}{2K}$ from the plume origin, therefore Equation (4) takes the form:

(5) $$\zeta \left(x,y\right)=\zeta_{\max}\left(\frac{x_{\max}}{x}\right)\exp\left[1-\left(\frac{y^{2}+z_{0}{}^{2}}{z_{0}{}^{2}}\right)\left(\frac{x_{\max}}{x}\right)\right].$$

**Figure 1:**
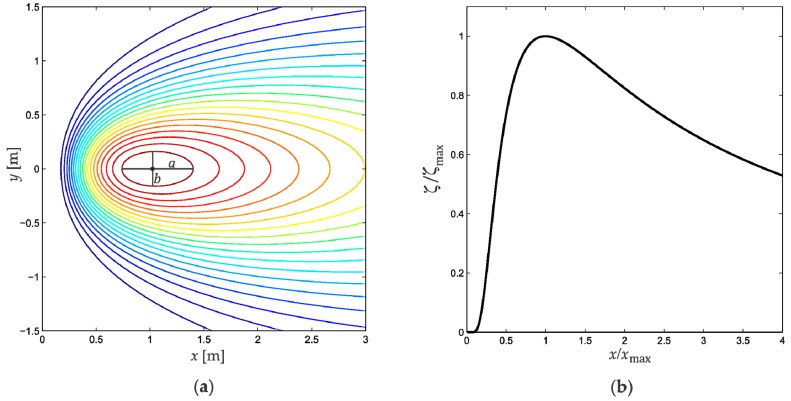
Gas source theoretical distribution: (**a**) Isoconcentration lines obtained by evaluation of Equation (5). The ovoid shape of the lines is determined by the wind, with the semi-major axis a aligned to the wind direction and with the semi-minor axis b aligned to cross-wind; (**b**) Normalized Gaussian concentration profile using Equation (6).

In practice, Equation (5) is a useful expression because it includes easily measurable quantities frequently found in this kind of plumes (maximum concentration and its position). In addition, the turbulent diffusion coefficient K and velocity u are not directly involved, so that it is not necessary to measure them. Equation (5) defines a concentration surface whose isoconcentration lines are shown in Figure 1a, with the values $\zeta_{\max}=1$ g/m^3^, $x_{\max}=1$ m and $z_{0}=0.7$ m. As expected, the maximum concentration is found along the *x*-axis at the $x_{\max}$ position. It is important to mention that both the maximum concentration and source positions are along the x-axis, which simplifies the experimental localization problem from 2D to 1D. Therefore, if we consider y=0 in Equation (5) the concentration profile along the x-axis is:

(6) $$\zeta \left(x\right)=\zeta_{\max}\left(\frac{x_{\max}}{x}\right)\exp\left[1-\left(\frac{x_{\max}}{x}\right)\right].$$

Figure 1b shows characteristic behavior of Equation (6), normalized in both concentration and length. In this figure the plume source is located at x=0 and the maximum concentration position at $x=x_{\max}$.

At first, one might think that the maximum concentration position must match the plume source. However, the maximum concentration position is displaced along the x-axis due to the sensor height $z_{0}$ and the wind velocity u, since $x_{\max}\propto uz_{0}^{2}$. It would not be surprising that tracking robots using *chemotaxis* algorithms have a singular behavior at this position. The maximum concentration position must match the plume source when the sensors are at soil level ($z_{0}=0$) or when there are not wind (u=0).

Another aspect that can be observed in Figure 1a is that the isoconcentration lines have an ovoid shape. These ovoids are concentric sharing as center the maximum concentration position $\zeta_{\max}$. Such a shape is determined by the wind, with the semi-major axis a coinciding with its direction. This information is experimentally relevant since it allows to determine the wind direction from concentration data, avoiding the use of specialized hardware.

### 2.2. Source Position from a Partial Distribution Profile

**Figure 2:**
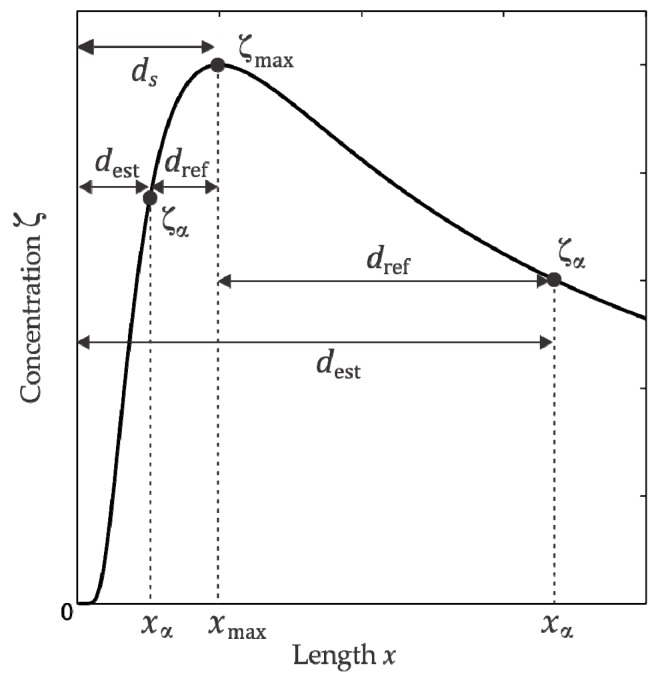
Determination of $d_{\mathrm{ref}}$ in function of the concentration $\zeta_{\alpha }$ on the left or on the right in relation to $\zeta_{\max}$. The position x=0 corresponds to the gas source location (unknown), while the maximum concentration is found at $x=x_{\max}$.

Due to the limited area of concentration mapping inside the room, it will be difficult to obtain a profile like the one shown in Figure 1b, instead just a piece of such profile is expected. Below, we will show that a partial profile along the x-axis containing the maximum concentration $\zeta_{\max}$ at $x_{\max}$ and any other concentration value $\zeta_{\alpha }$ at $x_{\alpha }$ (see Figure 2) will be sufficient to locate the plume source.

The distance $d_{\mathrm{ref}}$ in Figure 2 is easy to measure since it represents the segment between the maximum concentration point $x_{\max}$ and the point $x_{\alpha }$ on the x-axis. Depending if $d_{\mathrm{ref}}$ is to the left or right respect to $x_{\max}$, the cases $0<x_{\alpha }<x_{\max}$ and $x_{\alpha }>x_{\max}$ are distinguished, as Figure 2 shows. Therefore, the distance to the source on x-axis from the maximum concentration position $x_{\max}$ can be defined as:

(7) $$d_{s}=\{\begin{array}{ccc} d_{\mathrm{est}}+d_{\mathrm{ref}} & : & 0<x_{\alpha }<x_{\max}\\ d_{\mathrm{est}}-d_{\mathrm{ref}} & : & x_{\alpha }>x_{\max} \end{array}$$

with $d_{\mathrm{est}}$ to be determined. For this purpose, the rate $x_{p}=x_{\alpha }/x_{\max}$ is defined to get:

(8) $$d_{\mathrm{est}}=\{\begin{array}{ccc} +\left(\frac{x_{p}}{1-x_{p}}\right)d_{\mathrm{ref}} & : & 0<x_{\alpha }<x_{\max}\\ -\left(\frac{x_{p}}{1-x_{p}}\right)d_{\mathrm{ref}} & : & x_{\alpha }>x_{\max} \end{array}$$

where now *x_p_* is to be determined. Equation (6) is evaluated at $x_{\alpha }$ to find the $\zeta_{\alpha }$ value:

(9) $$\zeta_{\alpha }=\zeta \left(x_{\alpha }\right)=\zeta_{\max}\left(\frac{x_{\max}}{x_{\alpha }}\right)\exp\left(1-\frac{x_{\max}}{x_{\alpha }}\right),$$

which is reduced to:

(10) $$-\frac{\zeta_{p}}{e}=-\frac{1}{x_{p}}e^{-\frac{1}{x_{p}}},$$

with $\zeta_{p}=\zeta_{\alpha }/\zeta_{\max}$. Equation (10) is solved to find $x_{p}$ using the Lambert $W_{n}$ function [29]:

(11) $$x_{p}=-\frac{1}{W_{n}\left(-e^{-1}\zeta_{p}\right)}.$$

The Lambert $W_{n}$ function has two branches for real solutions, $W_{-1}$ and $W_{0}$. The n value is selected according to the $x_{\alpha }$ position: (a) for $0<x_{\alpha }<x_{\max}$ it is necessary that $W_{n}\left(-e^{-1}\zeta_{p}\right)<-1$, such condition is satisfied using $W_{-1}$; (b) for $x_{\alpha }>x_{\max}$ it is required that $-1<W_{n}\left(-e^{-1}\zeta_{p}\right)<0$, which is satisfied employing $W_{0}$. Substituting Equation (11) in Equation (8), $d_{\mathrm{est}}$ can be determined as:

(12) $$d_{\mathrm{est}}=\{\begin{array}{ccc} \left[\frac{-1}{W_{-1}\left(-e^{-1}\zeta_{p}\right)+1}\right]d_{\mathrm{ref}} & : & 0<x_{\alpha }<x_{\max}\\ \left[\frac{1}{W_{0}\left(-e^{-1}\zeta_{p}\right)+1}\right]d_{\mathrm{ref}} & : & x_{\alpha }>x_{\max} \end{array}.$$

Consequently, Equation (7) can be expressed according to:

(13) $$d_{s}=\{\begin{array}{ccc} d_{\mathrm{ref}}\left[1-\frac{1}{W_{-1}\left(-\frac{1}{e}\frac{\zeta_{\alpha }}{\zeta_{\max}}\right)+1}\right] & : & 0<x_{\alpha }<x_{\max}\\ d_{\mathrm{ref}}\left[\frac{1}{W_{0}\left(-\frac{1}{e}\frac{\zeta_{\alpha }}{\zeta_{\max}}\right)+1}-1\right] & : & x_{\alpha }>x_{\max} \end{array}.$$

These equations involves the maximum concentration and another concentration value along the x-axis, and their separation, quantities that can be easily measured.

## 3. Materials and Methods

### 3.1. Gas Sensors

It is common the use of ethanol [5], methanol [6], acetone [21] or gasoline [30] as agents to generate the plume. The constant in these pollutants is the storage capacity in liquid phase, volatile, heavier than air in gaseous state and easily detectable by commercial sensors. Other more aggressive contaminants have also been used, for instance, Russell et al. [2] used an ammonia solution in their *chemotaxis* study. We have chosen ethanol as the target gas in our experiments for safety, availability and its similar behavior to other popular fuels.

For the detection of ethanol gas, three metal-oxide sensors (MOX) model TGS2620 (Figaro Engineering Inc.; Mino, Osaka, Japan) were used. The main reasons for employing this sensor were its sensitivity to ethanol, availability, low cost and simple implementation. The characterization of our TGS2620 sensors was carried out in a hermetic chamber, generating known concentrations by injections of liquid ethanol and acquiring the response of the sensor to these stimuli. In this way, the discrete behavior of the sensors was obtained followed by a fitting to find the functional form. The manufacturer warns that these sensors in addition to responding to changes in concentration also respond to changes in temperature and humidity [31]. With the information provided in the datasheet we corrected the deviations of the readings due to variations in temperature and humidity.

While Equation (1) requires concentrations in units of mass/volume (ζ), in our experiments, the TGS2620 sensors operated under concentration data in parts per million per mole (ppm *n*/*n*). For ethanol, at a pressure of 0.78 atm (local pressure) and a temperature of 20 °C, we determined that the equivalence was 1.5 mg/m^3^ ↔ 1 ppm *n*/*n*. We denote concentrations in ppm *n*/*n* with the Latin letter C. Therefore, in our conditions:

(14) $$\zeta =\left(1.5\;\frac{\mathrm{mg}}{m^{3}\cdot \mathrm{ppm}}\right)C.$$

In the experimental development we will use C to denote concentration.

The TGS sensor family require a pre-heating time before their full operation [31]. We employed 20 min of preheating exposed to clean air before each experiment.

### 3.2. Data Acquisition, Robotic Platform and Fan

**Figure 3:**
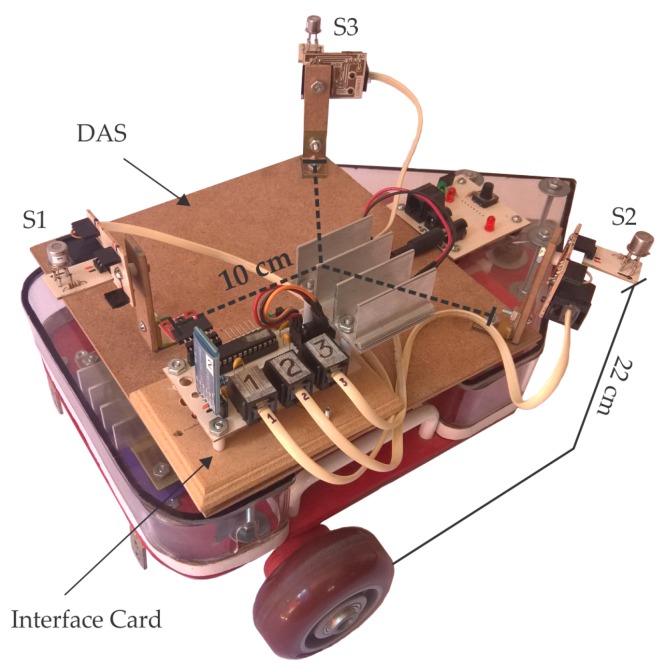
Robotic platform with the DAS. The three TGS2620 sensors are at a height $z_{0}=$ 22 cm.

We have designed three independent systems for (1) data acquisition, (2) autonomous mobility inside the room, and (3) generation of the plume. The first is a Data Acquisition System (DAS) which includes:

- Three TGS2620 sensors, together with the humidity and temperature sensor HIH6130 (Honeywell; Morris Plains, NJ, USA). The three TGS2620 sensors are placed facing up (22 cm height) at the vertices of an equilateral triangle as shown in Figure 3.
- An interface card as a link between the sensors and a computer, based on a PIC16F886 microcontroller (Microchip; Chandler, AZ, USA). It contains analog-digital converters for the TGS2620, it also implements the I^2^C communication protocol for the HIH6130 sensor and the UART protocol to transmit data to a computer via wireless.
- A computer in charge of sending commands to the interface card, as well as processing and storing the information received.

Upon a request from the computer, the interface card responds with a *data package* containing the voltage information of the three TGS2620 sensors, plus the temperature and humidity information provided by the HIH6130 sensor. The time from sending the command to receiving the data package was 200 ms (5 Hz), which is sufficient to notice any significant change in the signal of the TGS2620 [31]. Given the wireless characteristics of the DAS, it is not necessary for the computer to move with the rest of the system. The second system consists of a wireless autonomous robotic platform that provides mobility to the DAS. The platform is a mobile robot developed by our team using two DC motors in differential configuration. For the tests, a specific path was uploaded to the platform for an autonomous operation.

The plume was generated by a commercial 12 cm diameter fan and a 13 cm diameter container with 200 mL of ethanol. The fan was wirelessly operated by the computer and a microcontroller.

**Figure 4:**
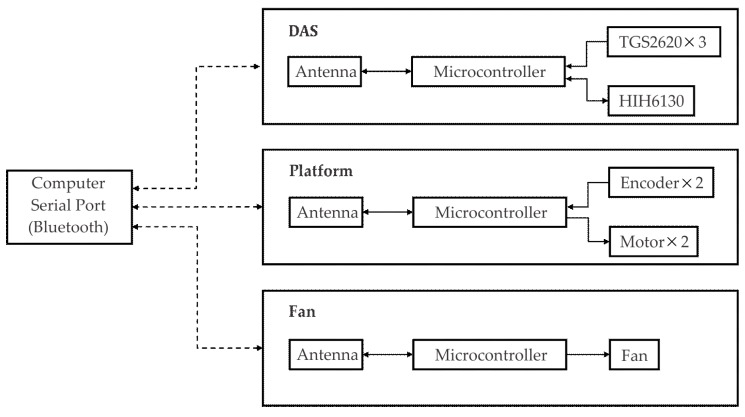
Interaction between the hardware.

The three systems, DAS-Platform-Fan, were designed to be independently or simultaneously operated by any computer with Bluetooth port (even with a smartphone). A schematic summary of the interaction between the elements is presented in Figure 4. All the information sent and received by the three systems was managed by a homemade software. Therefore, the administration of the fan, the platform path, the sensors data sampling, the storage and data processing, did not require a human operator.

### 3.3. Arena

**Figure 5:**
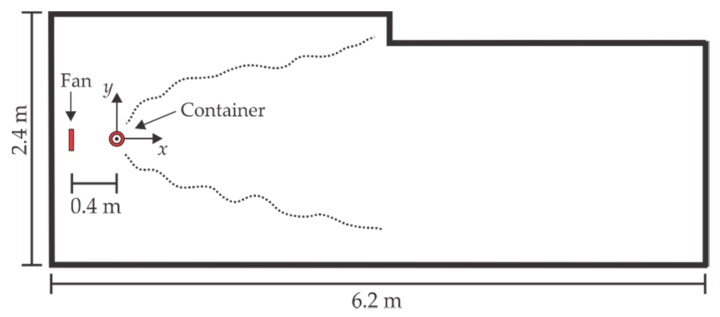
Room dimensions where the tests were performed.

The experiments were performed inside a 2.40 m × 6.20 m room as shown in Figure 5. The fan was located at a distance of 40 cm from the container center (x=−40 cm) generating a velocity above the container of 0.97 m/s, which is an acceptable value [10,32]. The fan-container arrangement produced a gas source with an approximately constant emission ratio of 0.46 mL/min. The environmental conditions in the room were 23 ± 2 °C and 35 ± 5% RH.

### 3.4. Measurements Order

We have established the order in our measurements to know:

- How is the wind velocity distribution generated by the fan inside the room. It is important to determine this distribution since the model assumes a constant wind velocity in one-direction.
- How is the gas concentration profile along *x*-axis for our fan-container arrangement. The gas experimental profile is the first indication of the possible application of the model.
- How is the concentration distribution inside the room generated by our fan-container arrangement at a $z_{0}$ height. If the concentration distribution is similar to a Gaussian plume at least in one region, then the model can be used.

## 4. Results and Discussion

### 4.1. Wind Velocity Distribution

The wind velocity distribution generated by the fan was measured to understand the behavior of our real gas source and was not used to estimate its position. This distribution was sampled with a hot-wire anemometer HT9829 (Xintest; Dongguan, Guangdong, China) with a measuring range of 0.1–25 m/s and a resolution of 0.01 m/s. During these measurements, the ethanol container was not present.

**Figure 6:**
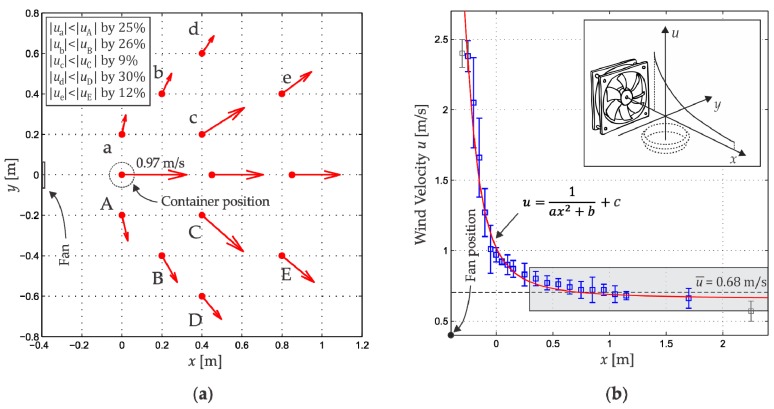
(**a**) Wind velocity distribution in the container vicinity. In the inset are presented the differences between uppercase and lowercase velocity magnitudes; (**b**) Wind velocity distribution along the x-axis.

Figure 6 shows the wind velocity measurements inside the room. The measurement time duration in all positions was one minute. Each arrow shown in Figure 6a is the average of 8 repetitions. We can observe that the velocity wind distribution is not unidirectional. Moreover, the velocity in the points in lowercase are on average 20% less than their equivalents in uppercase, that is, the distribution is asymmetric in magnitude. This velocity wind distribution is far from the model requirements. However, it is possible to observe that along the x-axis the wind moves in x-direction.

On the other hand, we performed measurements in 22 positions along the *x*-axis at a height of 6 cm (the fan axis) and spaced as shown in Figure 6b. Eight repetitions of these measurements were performed and their average values were plotted. According to the behavior of the wind velocity as a function of the distance, we consider that in the range 0.3<x<3.0 m this velocity is constant, and we assign it the average value $\bar{u}=0.68$ m/s.

With the above information we conclude that along the x-axis in the region 0.3<x<3.0 m the wind generated by the fan satisfies the model demands: unidirectional and uniform, in addition to flowing above the container. These characteristics indicated that a Gaussian concentration profile could be found in this region.

### 4.2. Ethanol Concentration Distribution Profile

**Figure 7:**
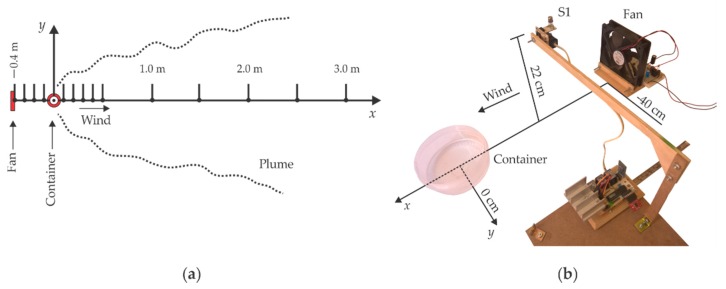
(**a**) Diagram of the 15 sampled concentration points along x-axis at height $z_{0}=22$ cm; (**b**) Setup to find the concentration profile at a height of $z_{0}=22$ cm by scanning a total distance of 3.4 m on the x-axis using Sensor 1. The fan-container arrangement that generates the gas plume can be observed.

To verify if our fan-container arrangement generated a Gaussian concentration profile, we measured the ethanol concentration at 15 different points along the x-axis using only Sensor 1 of the DAS, from 3.0 m to 0.5 m in 0.5 m steps and from 0.5 m to −0.4 m in 0.1 m steps, as indicated by Figure 7a. The DAS approached gradually to the fan on the x-axis as shown in Figure 7b. 

**Figure 8:**
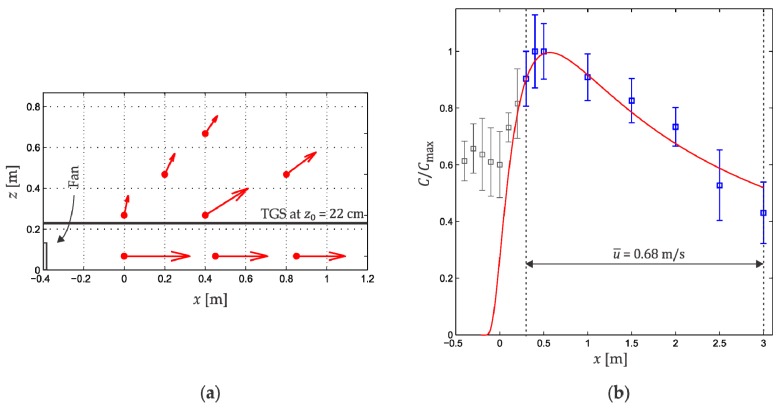
(**a**) Wind distribution in the xz plane; (**b**) Experimental concentration profile at a height $z_{0}=22$ cm.

The concentration sampling ratio was five data packages per second. The data were collected in each position for 2 min obtaining a total of 600 ethanol concentration readings, however only the last 300 concentration readings (most stable period) were used for the model calculations. Figure 8b presents the concentration profile measured for eight different approaches, so that, each point is the average of 2400 readings. The robotic platform was not used in these measurements.

If we consider a wind velocity distribution with axial symmetry around x-direction, we would obtain a profile as shown in Figure 8a. It is possible to observe that in the region between the fan and the vicinity of the container the wind conditions necessary for the Gaussian plume model at a height of 22 cm (TGS2620 sensor position) are not satisfied. The wind magnitude is not enough to avoid accumulation of ethanol in this region, as can be observed from the measurements shown in Figure 8b. It must be remembered that the Gaussian plume model assumes that behind the container the ethanol concentration is zero. On the other hand, the data in Figure 8a suggests that the wind direction is not valid between the fan and the vicinity of the container at 22 cm height. Therefore, the concentration values in this region do not fit the model, as observed in Figure 8b. Finally, we can say that in the interval 0.3<x<3.0 m the experimental profile fits the Gaussian concentration profile.

### 4.3. Ethanol Concentration Mapping Inside the Room and Locating the Source

At this point, we generate a plume using the fan-container arrangement considering the container position as unknown. This situation involves analyzing the ethanol concentration distribution inside the room to define a concentration profile and, using the information in Section 2.2, estimate the container position.

**Figure 9:**
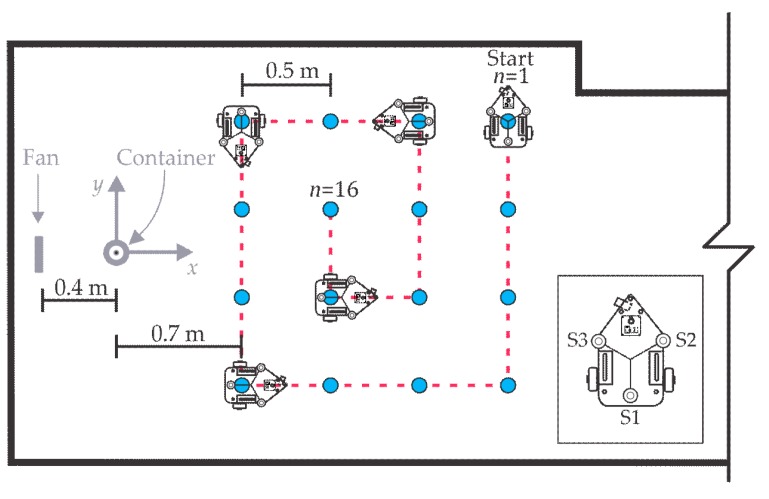
Room concentration mapping. The source location is unknown. The circles indicate the stops of the platform for data collection.

In order to estimate the ethanol gas distribution inside the room we uploaded a spiral path to the platform that covered most of the arena as shown by Figure 9. Each path was completed in 10.4 min. The platform performed 16 stops (circles) every 50 cm to collect data. The DAS collected 150 samples packages (Sensor 1, Sensor 2, Sensor 3, Temperature, Humidity) delaying 30 s at each n=1,…,16 positions resulting in 450 concentration readings at each stop, 150 samples per sensor:

$$S_{1,n}=\left(\begin{array}{c} a_{1,n}\\ \vdots \\ a_{150,n} \end{array}\right),S_{2,n}=\left(\begin{array}{c} b_{1,n}\\ \vdots \\ b_{150,n} \end{array}\right),\;S_{3,n}=\left(\begin{array}{c} c_{1,n}\\ \vdots \\ c_{150,n} \end{array}\right)$$

**Figure 10:**
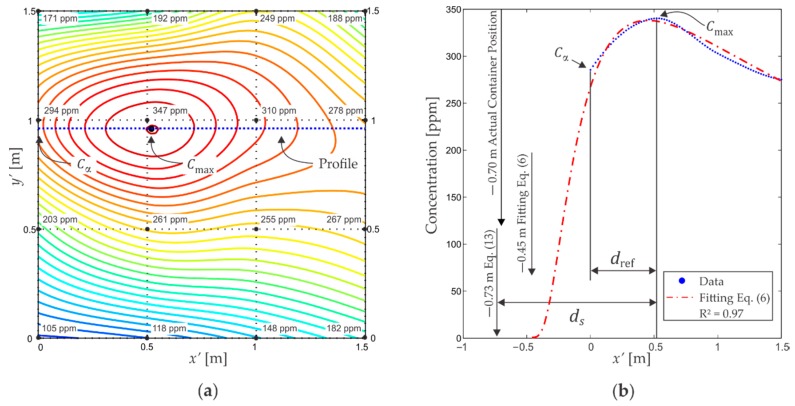

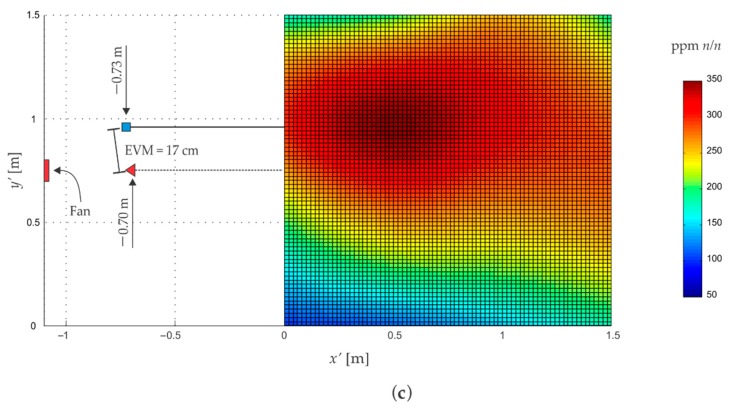
Concentration distribution data for a particular path. (**a**) Isoconcentration lines and plume symmetry axis. The average concentration values in each stop are also shown; (**b**) Dotted line, concentration profile along the symmetry axis obtained from interpolation. Dashdotted line, fitting using the whole measured profile; (**c**) Container position: the triangle shows the center of the container and the square indicates the estimated position according to Equation (13).

An average concentration value was assigned to each n position, such that:

(15) $$C_{n}=\frac{1}{150}\;\overset{150}{\underset{i=1}{\sum }}\left(a_{in}+b_{in}+c_{in}\right),$$

defining a concentration grid. We used the cubic interpolation method with the 16 concentrations distributed in a grid over the area, generating a concentration curve which covered the most of the arena. We performed 14 spiral paths under the same procedure, resulting in 14 concentration distributions, one for each path. These distributions had an average maximum concentration ${\bar{C}}_{\max}=327\pm 42$ ppm *n*/*n* and a minimum average concentration ${\bar{C}}_{\min}=148\pm 43$ ppm *n*/*n*. Figure 10 presents the results for one path, which will be used to describe the procedure to estimate the container position. The concentration curve obtained from interpolation is presented in Figure 10a using isoconcentration lines. According to Section 2.1, the symmetry axis of the plume must be parallel to the wind direction and to contain the maximum concentration point. Experimentally the problem of finding the symmetry axis is reduced to find in different ovoids the semi-major axis direction taking as a pivot the maximum concentration position which gives us a tendency of the wind direction. After that, we defined an auxiliary $x^{\prime }$-axis parallel to the symmetry one (dotted line), as shown in Figure 10a. The partial concentration profile was found along the symmetry axis, and for this path, the profile has the form shown in the Figure 10b.

To estimate the distance $d_{s}$ between the container position and the concentration maximum position along the symmetry axis, the maximum concentration $C_{\max}$ was located and a concentration $C_{\alpha }$ was chosen on the profile, therefore, the distance $d_{\mathrm{ref}}$ between these concentrations was determined as shown in Figure 10b, allowing the application of Equation (13). For this path and for this choice of concentration $C_{\alpha }$, a deviation of 3 cm was obtained between the estimation and the actual container position on the symmetry axis. Figure 10b also shows the fitting to the Equation (6) using all the profile points. For its part, this fitting establishes a deviation of 25 cm along the symmetry axis.

Figure 10c shows the concentration distribution in the mapping area together with the container and the fan locations. The center of the container is presented as a triangle and the estimated location by applying Equation (13) is presented as a square. The *error vector magnitude* (EVM) is the deviation between the estimation and the actual container position in the area. In this case such deviation was 17 cm.

As stated in Section 2.2 about the use of Equation (13), $C_{\alpha }$ can take any value along the concentration profile, however experimentally the container estimated position was affected by the selection of $C_{\alpha }$. To find the best estimation, each of the concentration values that constitute the profile (except $C_{\max}$) was introduced as parameter $C_{\alpha }$ in Equation (13).

**Figure 11:**
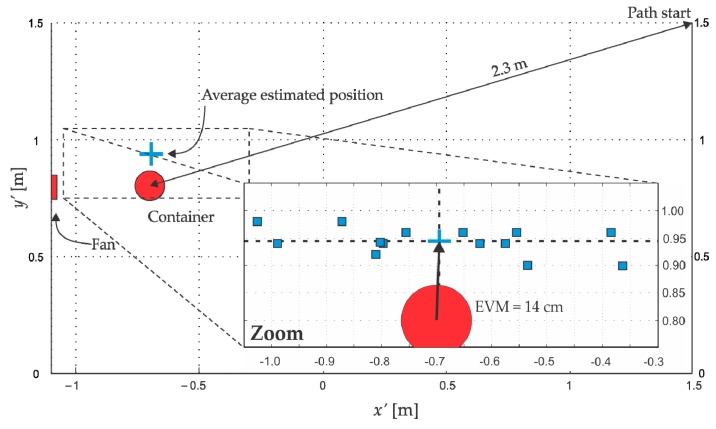
Estimated positions of the container for the 14 paths using Equation (13) and $C_{\alpha }=0.9C_{\max}$ in the region of steeper slope. The cross represents the average estimated position. Container dimensions in proportion.

As shown in Figure 2, this equation distinguishes two regions, one to the left of $C_{\max}$ characterized by a profile with a steep slope and another region to the right, with a less steep slope. The best estimation of the container position was obtained when $C_{\alpha }=0.9C_{\max}$ in the region of steeper slope (left), which is found between the container and $C_{\max}$. The selection of $C_{\alpha }=0.9C_{\max}$ is in agreement with the profile shown in Figure 8b where the first value not affected by the ethanol accumulation was (30 cm, $0.9C_{\max}$). In Figure 11, the container estimated positions via Equation (13) using $C_{\alpha }=0.9C_{\max}$ for each of the 14 paths are shown as squares and the average estimated position is presented as a cross. The EVM between the average estimated position and the actual container position was 14 cm.

In Figure 11, all estimations (squares) are deviated from the actual container position in the $y^{\prime }$-direction since the maximum concentrations are also deviated, as can be observed in the distribution in Figure 10a where a single path is shown. We consider that this effect is due to the nonuniform wind intensity (see Figure 6a). It is probable that this situation provoked the maxima concentrations to move toward a less drag area causing biased estimations. Therefore, this fact can be to assumed as a systematic error that affects only in our circumstances. Possibly the EVM would tend to zero for an average estimated position obtained by a symmetric wind velocity distribution.

The Gaussian concentration profile has a characteristic shape as seen in Figure 1a. For the best implementation of Equation (13), the partial experimental profile must fit to the theoretical profile where the steepest slope would indicate the direction towards where the source is located. A profile different from that shown in Figure 1a would not allow the use of Equation (13).

Regarding the quality of the estimation, our average estimated position had an EVM of 14 cm, which represents the 6.1% of the shortest path between the initial position *Start* (Figure 11) and the container position. Marques et al. [6] considered their experiment completed when their robot entered a circle of 50 cm radius (10%) with its source in the center, while Ishida et al. [5] have estimated the source position with an EVM of 6.4 cm (6.4%), which are very satisfactory results. It is worth to mention that in our localization process we do not use anemometric systems.

## 5. Conclusions

An application of the Gaussian plume model in an empty room was shown in this article. The main motivation was to use the model to locate the plume source. This work involved the theoretical model adaptation, the study of the wind distribution generated by our fan, the robotic system implementation for mobility and data acquisition, and the concentration distribution mapping. In addition, a mathematical expression was derived to estimate the source position which uses only two concentration values of the whole distribution.

The results showed that, inside a room, the Gaussian plume model partially describes the experimental plume generated by a conventional fan and a container with liquid ethanol, despite having a quasi-uniform and quasi-unidirectional wind distribution.

Even though a mapping of the arena is necessary, by using the isoconcentration lines we showed that the sufficient information to localize the gas source is contained in the symmetry axis, which reduces the problem from 2D to 1D.

The model application allowed to estimate the source position using only gas sensors. The localization time was 10.4 min and the average estimated position had a deviation of 14 cm (6.1%) in relation to the actual source position. Such values are comparable to similar studies that use, in addition to gas sensors, expensive anemometric systems.
